# Mendelian randomization of circulating proteome identifies actionable targets in heart failure

**DOI:** 10.1186/s12864-022-08811-2

**Published:** 2022-08-13

**Authors:** Louis-Hippolyte Minvielle Moncla, Samuel Mathieu, Mame Sokhna Sylla, Yohan Bossé, Sébastien Thériault, Benoit J. Arsenault, Patrick Mathieu

**Affiliations:** 1 Genomic Medecine and Molecular Epidemiology Laboratory, Quebec Heart and Lung Institute, Laval University, Quebec, G1V-4G5 Canada; 2 Department of Molecular Medicine, Laval University, Quebec, Canada; 3 Department of Molecular Biology, Medical Biochemistry and Pathology, Laval University, Quebec, Canada; 4 Department of Medicine, Laval University, Quebec, Canada; 5 Department of Surgery, Laval University, Quebec, Canada

**Keywords:** Heart failure, Mendelian randomization, Blood protein, Network, Pathway, Druggable genome

## Abstract

**Background:**

Heart failure (HF) is a prevalent cause of mortality and morbidity. The molecular drivers of HF are still largely unknown.

**Results:**

We aimed to identify circulating proteins causally associated with HF by leveraging genome-wide genetic association data for HF including 47,309 cases and 930,014 controls. We performed two-sample Mendelian randomization (MR) with multiple *cis* instruments as well as network and enrichment analysis using data from blood protein quantitative trait loci (pQTL) (2,965 blood proteins) measured in 3,301 individuals. Nineteen blood proteins were causally associated with HF, were not subject to reverse causality and were enriched in ligand-receptor and glycosylation molecules. Network pathway analysis of the blood proteins showed enrichment in NF-kappa B, TGF beta, lipid in atherosclerosis and fluid shear stress. Cross-phenotype analysis of HF identified genetic overlap with cardiovascular drugs, myocardial infarction, parental longevity and low-density cholesterol. Multi-trait MR identified causal associations between HF-associated blood proteins and cardiovascular outcomes. Multivariable MR showed that association of BAG3, MIF and APOA5 with HF were mediated by the blood pressure and coronary artery disease. According to the directional effect and biological action, 7 blood proteins are targets of existing drugs or are tractable for the development of novel therapeutics. Among the pathways, sialyl Lewis x and the activin type II receptor are potential druggable candidates.

**Conclusions:**

Integrative MR analyses of the blood proteins identified causally-associated proteins with HF and revealed pleiotropy of the blood proteome with cardiovascular risk factors. Some of the proteins or pathway related mechanisms could be targeted as novel treatment approach in HF.

**Supplementary Information:**

The online version contains supplementary material available at 10.1186/s12864-022-08811-2.

## Background

Despite significant advances in the treatment of heart failure (HF) in the last decade, the life expectancy for patients with this condition is still limited [1]. Only a small fraction of HF cases is related to a monogenic cardiomyopathy [2]. A recent genome-wide association study (GWAS) leveraging 47,309 cases and 930,014 controls has identified 11 loci associated to HF [3]. HF is characterized by an altered left ventricular (LV) function and may result from different causes. Notably, coronary artery disease (CAD), high blood pressure, atrial fibrillation and some other cardiometabolic risk factors are associated with the development of HF [4]. The molecular processes and key factors promoting the development of HF are still largely unknown. The identifications of molecules promoting the development of HF could lead to novel therapy. Epidemiological studies measuring biomarkers are subject to bias and reverse causality [5]. Hence, only a small proportion of trials are successful and lead to new licensed drugs [6, 7].

Genetic association data provide a rich resource to identify molecules and pathways involved in the development of disorders. Gene variants acting in *cis* and associated with intermediate phenotypes such as the expression of genes or the level of proteins in circulation can be leveraged as instrumental variables (IVs) in Mendelian randomization (MR) [8]. Since alleles are randomly allocated before the development of outcomes, MR technique is not prone to reverse causality [9]. Hence, the use of multiple IVs in MR is a robust method to evaluate causal associations. Studies have underscored that molecular targets supported by genetics have a higher chance for being licensed [10].

Disorders are complex systems characterized by the interaction of several molecules. The assessment of complex system in network is a non-biased method to probe pathways and to prioritize molecules [11, 12]. Studies have consistently underscored that molecules highly connected in network, often referred to as hubs, are enriched in signaling pathways and drug targets [13]. Herein, we implemented an integrative approach to identify causally associated blood proteins with HF and we performed network and pathway analyses to prioritize molecules and find novel druggable candidates.

## Results

### Mendelian randomization of blood proteins in heart failure

**Fig. 1:**
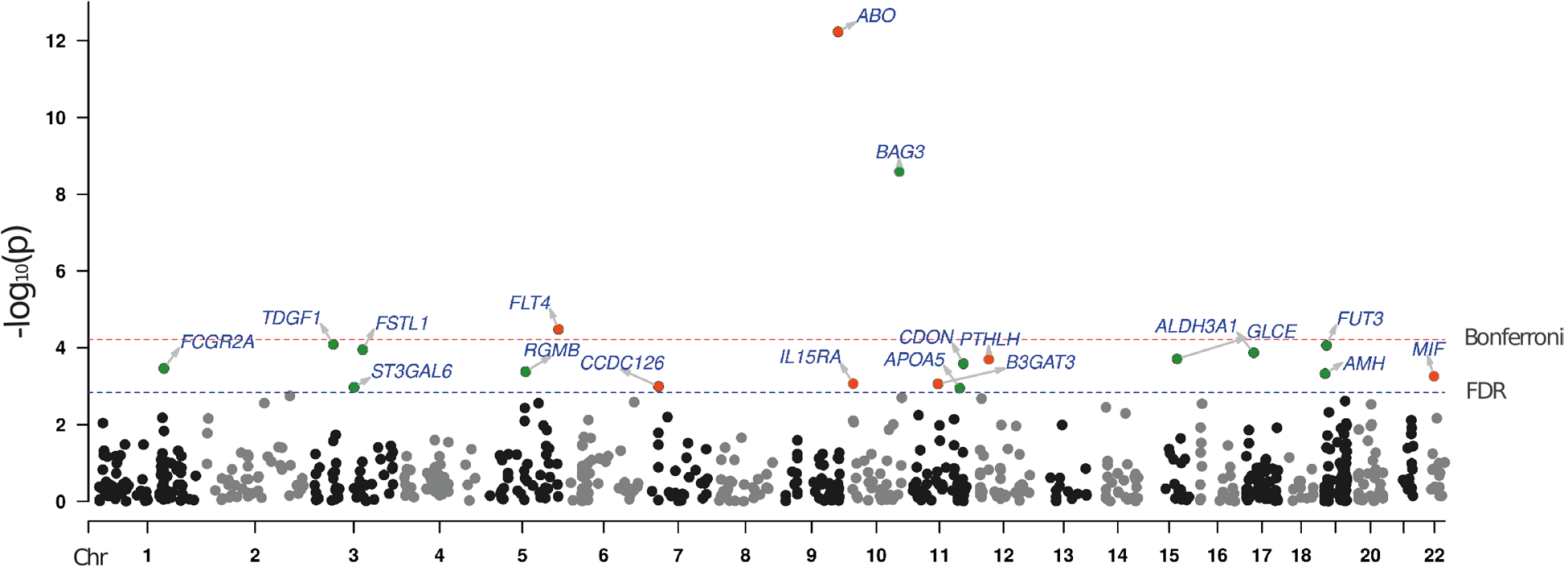
Identification of blood proteins potentially implicated in HF. Manhattan plot depicting blood proteins associated with heart failure (HF) in *cis*-MR analysis. The localization of the gene encoding the blood protein is represented on the x-axis, whereas the y-axis represents the -log_10_*P* value for the association in MR. Red and blue dashed lines are the Bonferroni and FDR 5% threshold values respectively. Red dots are genes positively associated with the development of HF, and green dots are genes negatively associated with the development of HF

We conducted a two-sample MR analysis to identify causally associated blood proteins with HF. Summary level data from INTERVAL [14], a study including pQTLs for 2,965 different blood proteins measured in 3,301 individuals, were leveraged to identify *cis*-acting gene variants as instrumental variables (IVs). A minimum of 3 independent (r2 < 0.1) gene variants within a window of 500 kb were selected to identify IVs (*P* value < 1E-03) for the blood proteins (exposure). GWAS data from the Heart Failure Molecular Epidemiology for Therapeutic Targets Consortium (HERMES) [3], a meta-analysis including 47,309 HF cases and 930,014 controls of European ancestry, were assessed as the outcome. There were enough instruments to perform 822 *cis*-MR analyses. In inverse variance weighted (IVW) MR, we identified at a false discovery rate (FDR) of 5% nineteen blood proteins that were significantly associated with HF (ABO, BAG3, FLT4, TDGF1, FUT3, FSTL1, ALDH3A1, GLCE, PTHLH, CDON, FCGR2A, RGMB, AMH, MIF, IL15RA, B3GAT3, CCDC126, ST3GAL6, APOA5) (Fig. 1) (Suppl. Table 1 and Suppl. Table 2). In order to check for weak instruments, we calculated F-statistic [15] for all identified instruments for each protein significantly associated with HF. The F-statistic was > 10 for each variant confirming the validity of our selected IVs [15–17] (Suppl. Table 1). Among those proteins, (OR per 1 SD) ABO (OR: 1.03, 95%CI: 1.02–1.04, P_IVW_ = 5.89E-13), BAG3 (OR: 0.79, 95%CI: 0.74–0.85, P_IVW_ = 2.59E-09) and FLT4 (OR: 1.08, 95%CI: 1.04–1.12, P_IVW_ = 3.34E-05) were significant after a Bonferroni correction (*P* < 6.08E-05, 0.05/822). We carried out the Cochran’s Q and Egger intercept tests to detect horizontal pleiotropy [18, 19]. Both the Cochran’s Q and intercept tests did not reveal heterogeneity or horizontal pleiotropy for the nineteen blood proteins (Suppl. Table 1). Thus, the nineteen blood proteins (FDR < 0.05) were considered as causal molecular candidates for downstream analyses. Among the causal candidates, 7 (ABO, FLT4, PTHLH, MIF, IL15RA, B3GAT3, CCDC126) were positively associated with HF, whereas 12 (BAG3, TDGF1, FUT3, FSTL1, ALDH3A1, GLCE, CDON, FCGR2A, RGMB, AMH, ST3GAL6, APOA5) were negatively associated with the risk of HF. The blood proteins with the largest negative and positive effect sizes on HF were BAG3 (OR: 0.79, 95%CI: 0.74–0.85, P_IVW_ = 2.59E-09) and MIF (OR: 1.19, 95%CI: 1.08–1.32, P_IVW_ = 5.53E-04), respectively.

As MR is subject to pleiotropy of the IVs, we performed several sensitivity analyses. In weighted median MR, which is robust to invalid instruments [20] (variants with horizontal pleiotropy), 17 blood proteins (ABO, BAG3, CDON, APOA5, CCDC126, FLT4, IL15RA, ALDH3A1, PTHLH, RGMB, AMH, GLCE, TDGF1, FSTL1, FCGR2A, B3GAT3, MIF) remained significantly associated with HF (Suppl. Table 1). The directional effects were concordant between weighted median MR and IVW MR. As an additional measure we implemented MR by using *cis*-IVs with a more stringent *P* value (*P* < 1E-05). By using this approach, there were enough instruments to perform multiple instruments MR for 12 causal candidate proteins. In MR, the 12 proteins (ABO, TFGF1, GLCE, CCDC126, IL15RA, FCGR2A, CDON, FUT3, ST3GAL6, B3GAT3, ALDH3A1, FLT4) remained significantly associated with HF (FDR < 0.05) with concordant directional effect (Suppl. Table 3). Among the remaining 7 proteins, 5 had one available instrument with a *P* value < 1E-05, for which we performed MR with the Wald ratio. MR with the Wald ratio showed that 4 proteins (BAG3, PTHLH, RGMB, APOA5) were significantly (P_Wald_ test < 0.05) associated with HF and again with concordant directional effects (Suppl. Table 4). Hence, these analyses showed that 16 blood proteins out of 19 were replicated by using a more stringent selection of IVs. We also conducted reverse MR analysis as an additional sensitivity measure. We selected 11 genome-wide significant instruments at HF risk loci. In reverse MR analysis, we found no significant association (FDR > 5% for the nineteen causal candidate proteins) (Suppl. Table 5). These data indicate that the causal candidate blood proteins were not subject to reverse causality.

Additionally, we conducted a replication two-sample MR analysis by leveraging as exposure the data from the deCODE study [21]. This study included pQTLs for 4,719 blood proteins measured in 35,559 Icelanders of European ancestry. Enough instruments were available to perform 17 *cis*-MR analysis (*P* value IVs < 1E-03) (Suppl. Table 6). Among the 17 candidate blood proteins available in the deCODE study, 14 were replicated at FDR < 5% (ABO, GLCE, IL15RA, RGMB, FSTL1, CDON, ALDH3A1, ST3GAL6, TDGF1, FUT3, CCDC126, FCGR2A, PTHLH, B3GAT3) (Suppl. Table 6). By using IVs with a *P* value < 1E-05, both multiple instruments MR and the Wald ratio showed that 16 proteins (ABO, FLT4, GLCE, IL15RA, TDGF1, B3GAT3, RGMB, FSTL1, ALDH3A1, CDON, ST3GAL6, FUT3, FCGR2A, CCDC126, PTHLH, MIF) were replicated in deCODE (Suppl. Table 7 and Suppl. Table 8). For the replicated blood proteins, the directional effects were concordant between deCODE and INTERVAL.

### Enrichment and network pathway analysis

We aimed to identify the functional and pathway enrichments of candidate causal blood proteins. Among the causal candidates, 5 blood proteins (ABO, FUT3, GLCE, B3GAT3 and ST3GAL6) were classified as molecules involved in glycosylation in the Comprehensive GlycoEnzyme Database (GlycoEnzDB) (fold-enrichment 13.1, *P* = 1.40E-06, hypergeometric test). Of note, FUT3 (OR: 0.97, 95%CI: 0.96–0.98, P_IVW_ = 8.68E-05) and ST3GAL6 (OR: 0.97, 95%CI: 0.96–0.99, P_IVW_ = 1.06E-03) were both negatively associated with the risk of HF. FUT3 and ST3GAL6 are key enzymes leading to the generation of sialyl Lewis x, a glycan moiety decorating membrane and circulating proteins [22, 23] (Suppl. Figure 1). We next hypothesized that some of the causal candidate proteins may be involved in different ligand-receptor interactions. By using a comprehensive repository of ligand-receptor interactions reported by Shao et al. [24], we found that blood proteins associated with HF in MR were enriched in ligand and receptors (fold-enrichment 5.7, *P* = 4.0E-07, hypergeometric test). These molecules may contribute to 43 different ligand-receptor pairs (Suppl. Table 9). The 43 ligand-receptor pairs were enriched in Gene Ontology (GO) (molecular function) for transmembrane receptor protein serine/threonine kinase activity (*P* = 3.68E-12), G protein-coupled receptor activity (*P* = 1.08E-10), patched binding (*P* = 5.43E-07), activin-activated receptor activity (*P* = 1.30E-06) and transforming growth factor beta-activated receptor activity (*P* = 3.38E-06) (Suppl. Figure 2) (Suppl. Table 10).

**Fig. 2:**
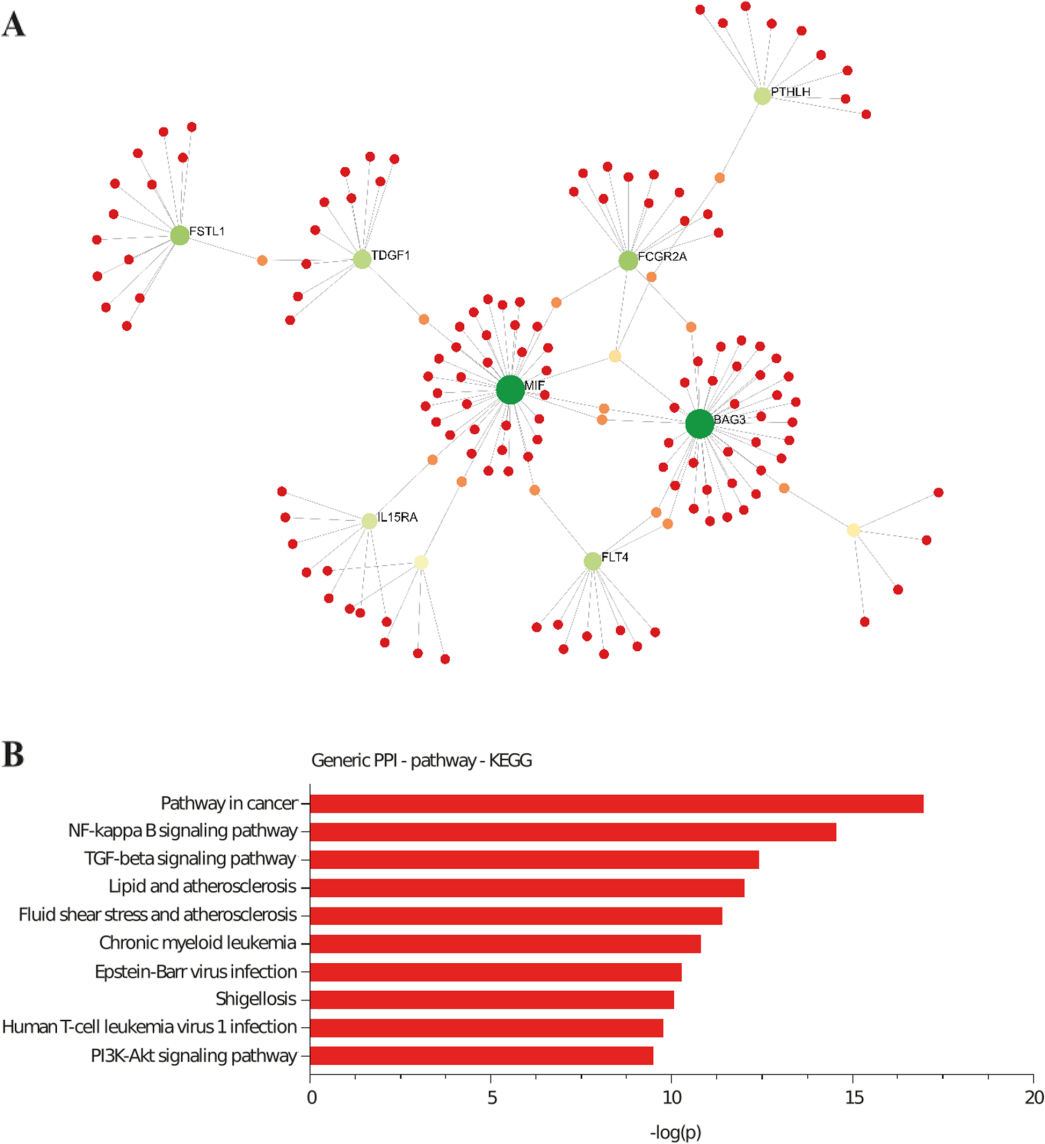
Network and enrichment pathway analysis of causal blood protein candidates. A) HF causal blood protein candidates were used as seeds to generate a protein interaction network inferred from InnateDB [25] (database of 19,800 curated proteins interactions). B) Pathway enrichment analysis of the network by using the Kyoto Encyclopedia of Genes and Genomes (KEGG) [26]

We next performed a pathway analysis by using a network approach. Protein interaction data from InnateDB, which includes more than 19,800 curated protein interactions, was leveraged to infer a blood protein network [25]. The nineteen causal candidate proteins were used as seeds to generate a network including 155 nodes (proteins) and 160 edges (interactions) (Fig. 2A) (Suppl. Table 11). The causally associated blood proteins were overrepresented in the nodes (proteins) with the highest degree (≥ 90^th^ percentile) (fold-enrichment 3.6, *P* = 7.26E-06, hypergeometric test). The top nodes (proteins) acting as hub molecules include MIF, BAG3, FSTL1, FCGR2A, TDGF1, FLT4, PTHLH, IL15RA, AMH and ALDH3A1. We interrogated the Kyoto Encyclopedia of Genes and Genomes (KEGG) [26] to perform a pathway enrichment analysis of the network. The highest enrichments were pathways in cancer (*P* = 1.03E-17), NF-kappa B signaling (*P* = 2.99E-13), TGF-beta signaling (*P* = 3.83E-13), lipid and atherogenesis (*P* = 9.53E-13) as well as fluid shear stress and atherosclerosis (*P* = 9.96E-12) (Fig. 2B) (Suppl. Table 12).

### Cross-phenotype analysis

**Fig. 3:**
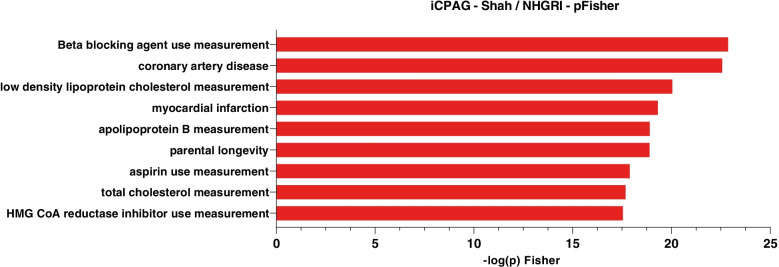
Cross-phenotype analysis. Cross-phenotype association analysis of HF performed by using the summary statistics data of GWAS from HERMES and the interactive cross-phenotype analysis of GWAS database (iCPAG), which includes data from the NHGRI-EBI GWAS catalog. Significance of traits was determined by the Fisher exact test

Cross-phenotype association analysis were performed for the genetic association data of HF by using the interactive cross-phenotype analysis of GWAS database (iCPAG) [27] which provides enrichment and similarity metrics between traits by using an exhaustive list of ancestry LD-specific association data from the NHGRI-EBI GWAS catalog [28]. After a Bonferroni correction, this analysis showed that 75 disorders and traits were significantly associated to HF (Suppl. Table 13). Figure 3 shows the highest enrichments between HF and traits-disorders. According to iCPAG, the highest enrichments were for beta blocking agent use (*P* = 1.36E-24), coronary artery disease (*P* = 2.70E-23), low-density cholesterol (*P* = 9.09E-21), myocardial infarction (*P* = 4.87E-20), apolipoprotein B (*P* = 1.24E-19) and parental longevity (*P* = 1.28E-19).

### Multi-trait and multivariable MR analyses

**Fig. 4:**
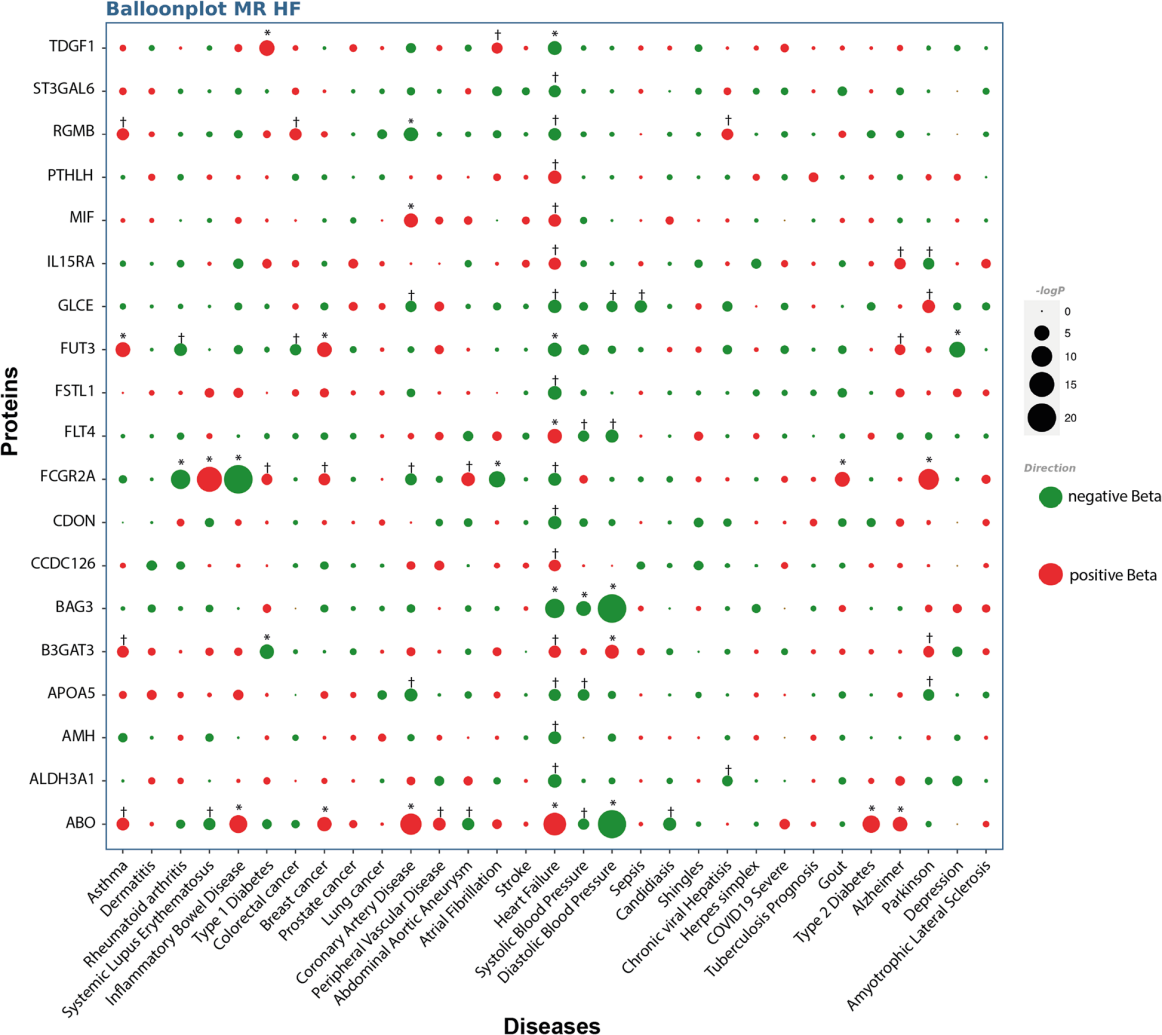
Multi-trait MR. Balloon plot illustrating the multi-trait MR analysis of the 19 blood proteins as exposures, and the 31 diseases and traits as outcomes. Red and green indicates positive and negative directional effects respectively. * Indicates significance at a Bonferroni threshold; † indicates significance at FDR 5% threshold

Considering the cross-phenotype analysis showing a genetic overlap between HF and several cardiovascular disorder related traits, we performed a multi-trait MR analysis for the nineteen causal blood candidate proteins. We leveraged 31 different GWAS covering 7 disease categories (atopic, autoimmune, cancer, cardiovascular, infectious, metabolic and neurologic) as outcomes (Suppl. Table 14). Figure 4 illustrates the MR analysis including the directional effect and the significance (-logP) between the nineteen blood proteins (exposure) and the different disorder-traits (outcomes). Some blood proteins such as ABO and FCGR2A show significant association with several traits often with opposite directional effects (antagonistic pleiotropy) with HF. However, some proteins such as BAG3, MIF and APOA5 show concordant associations between HF, the blood pressure (BAG3) and coronary artery disease (MIF and APOA5). BAG3 is significantly and negatively associated with systolic and diastolic blood pressure, whereas MIF and APOA5 are associated with coronary artery disease. We performed mediation analysis by using multivariable MR corrected for the exposure to cardiovascular traits. Following the correction for the diastolic blood pressure, the association between BAG3 and HF was not significant (Suppl. Table 15). Also, after correction for coronary artery disease the associations between MIF and APOA5 with HF were no longer significant (Suppl. Table 15). Taken together, these data suggest that, at least in part, the protective effect of circulating BAG3 is mediated by a reduction of the diastolic blood pressure. On the other hand, the impact of MIF and APOA5 on HF are likely mediated by the risk of coronary artery disease, a leading cause of HF [29, 30].

### Drug target analysis

We leveraged several resources to carry out a drug target analysis. We investigated the blood proteins in order to document if they represent targets for licensed, in-development small molecules or biologics. In the Therapeutic Target Database (TTD) [31], 6 blood proteins (ABO, FLT4, FCGR2A, AMH, MIF, IL15RA) are reported as either clinical trial target or successful targets (Suppl. Table 16). In the Drug Gene Interaction Database (DGIdb) [32], a total of 111 drug-target pairs were reported for FLT4, ALDH3A1, PTHLH, FCGR2A, AMH, MIF, IL15RA and APOA5 (Suppl. Table 17). Several kinase inhibitors targeting FLT4, a blood protein positively associated with the risk of HF, are approved for the treatment of cancer. In the Open Targets database [33, 34], MIF, PTHLH, FLT4 and TDGF1 were reported as targets of approved and in-development drugs as well as antibodies (Suppl. Table 18). In the blood, MIF is positively associated with the risk of HF and is a target for Imalumab and Iguratimod, respectively an antibody and a small molecule inhibitor. Iguratimod is licensed in Japan for the treatment of rheumatoid arthritis [35], whereas Imalumab is a phase 1 monoclonal antibody destined for patients with solid tumors [36]. According to Open Targets, 15 blood proteins (MIF, CCDC126, IL15RA, FCGR2A, CDON, ALDH3A1, FSTL1, APOA5, AMH, BAG3, B3GAT3, ST3GAL6, FUT3, GLCE, RGMB) are deemed tractable for the development of antibodies (Suppl. Table 18). Taken together, these data suggest that according to the directional effect MIF, FLT4, PTHLH, ABO, CCDC126, IL15RA, and B3GAT3 (blood proteins positively associated with HF) are potential targets for HF as they are the object of approved, in-development inhibitors or deemed tractable for the development of novel inhibitors (antibodies).

## Discussion

In this work, we undertook a MR analysis of the circulating proteome to identify circulating proteins causally associated with HF. A comprehensive analysis of the blood proteome identified, by using multiple *cis-*acting variants as IVs, nineteen proteins causally associated with HF. Causal candidate proteins were enriched in glycosylation and ligand-receptor molecules. A blood protein network showed that causal candidates were enriched as hub molecules involved in pathways related to NF-kappa B, TGF-beta and atherogenesis. Cross-phenotype and multi-trait MR showed that some blood proteins associated with HF were pleiotropic and involved in cardiovascular traits. The assessment of the druggable genome identified causal candidates for which drug repurposing or drug development could lead to novel therapies.

The present MR analysis identified several novel genes associated with HF. In this regard, among the candidate molecules only ABO and BAG3 have been previously associated with HF. Among the causal blood candidate proteins, BAG3 (OR: 0.79, 95%CI: 0.74–0.85, P_IVW_ = 2.59E-09) and MIF (OR: 1.19, 95%CI: 1.08–1.32, P_IVW_ = 5.53E-04) had the highest effect size in MR analysis. Mutations in BAG3 have been associated with dominant forms of myopathy affecting skeletal muscle and the heart [37]. The role of intracellular BAG3 has been intensively investigated as the protein binds to heat shock proteins and control protein folding and aggregation. Herein, we documented, to our knowledge, for the first time that secreted and circulating BAG3 may be protective on the risk of HF. A previous study showed that BAG3 administered to rodents reduced the blood pressure through a nitric oxide pathway [38]. The putative receptor whereby extracellular BAG3 reduced the blood pressure is presently unknown. These experimental data are supported by the present results, which showed in mediation analysis that the protective effect of circulating BAG3 on HF was, at least in part, mediated by a reduction of the diastolic blood pressure. Functional follow-up studies are needed to tease apart the mechanism whereby circulating BAG3 regulates the blood pressure and the risk of HF. *MIF* encodes the macrophage migration inhibitory factor, a pro-inflammatory molecule involved in rheumatoid arthritis [39]. Results of our MR analysis indicated that MIF was positively associated with the risk of HF. Multivariable analysis suggested that MIF promoted HF through the risk of CAD.

The circulating proteins related to the risk of HF were highly enriched in molecules involved in glycosylation (fold-enrichment 13.1, *P* = 1.40E-06). Notably, FUT3 (OR: 0.97, 95%CI: 0.96–0.98, P_IVW_ = 8.68E-05) and ST3GAL6 (OR: 0.97, 95%CI: 0.96–0.99, P_IVW_ = 1.06E-03) were negatively associated with the risk of HF. *FUT3* and *ST3GAL6* encode for fucosyl transferase 3 and ST3 beta-galactoside alpha-2,3-sialyltransferase 6, respectively. They are involved in the synthesis of sialyl Lewis X (Siaα2,3Galβ1,4[Fucα1,3] GlcNAc), a glycan moeity involved in cell adhesion and the recruitment of inflammatory cells (Suppl. Figure 1). Experimental evidence suggests that circulating glycosyltransferase promotes extracellular glycosylation including the synthesis of sialyl Lewis x [40]. These data suggest that the synthesis of sialyl Lewis X in the blood could be protective in HF possibly by acting as a decoy factor that limit leukocyte adhesion. Consistent with this hypothesis, systemic administration of a sialyl Lewis x analogue in a large animal model of cardiac arrest decreased the recruitment of inflammatory cells to the myocardium and preserved the myocardial function [41]. Further work is needed to address the potential role of glycosylation in HF.

HF-associated causal proteome was enriched in ligands and receptors. Ligand-receptor pairs were overrepresented by serine/threonine kinase, G protein-coupled receptor, patched binding, activin-activated receptor binding and transforming growth factor beta-activated receptor activity. Blood IL15RA (OR: 1.02, 95%CI: 1.01–1.04, P_IVW_ = 8.60E-04) concentrations were positively associated with the risk of HF. *IL15RA* encodes for the IL15R alpha-chain, which is secreted through a protease-dependent process and binds to IL15 [42, 43]. Secreted receptors may function as natural antagonist or agonist for their ligands and thus exert an important control over many biological processes. Contradicting reports for secreted IL15RA suggest that it may either antagonize or promote IL15 signaling [44, 45]. In a mouse model of myocardial infarction, the administration of IL15 improved cell death and LV function [46]. Inquiry into the function of IL15RA-IL15 on the myocardial function could help design new therapies. *TDGF1* encodes for teratocarcinoma-derived growth factor 1 (also known as CRIPTO), which is acting as natural antagonist for activin receptor ActRII [47, 48]. Blood TDGF1 (OR: 0.98, 95%CI: 0.97–0.99, P_IVW_ = 8.20E-5) concentrations were negatively associated with the risk of HF suggesting that inhibition of the activin pathway might reduce the risk of HF. Consistent with the present data, activin-mediated signaling (ActRII) in cardiomyocyte promoted the degradation of sarcoplasmic reticulum Ca2 + ATPase (ATP2A2 also known as SERCA2), an important regulator of cardiac contractility, and the development of aging-related HF in a rodent model [49]. Among the other blood proteins acting as receptors, CDON (OR: 0.95, 95%CI: 0.93–0.98, *P* = 2.59E-04) is involved in hedgehog (HH) signaling [50], a pathway playing a role in cardiac regeneration [51, 52], whereas AMH (OR: 0.82, 95%CI: 0.74–0.91, *P* = 4.71E-04) binds to AMHR2 and is related to the TGF-beta pathway [53]. *AMH* encodes for the anti-Müllerian hormone. Lower level of this hormone have been associated with increased cardiovascular risk in women [54].

Some of the causal blood protein candidates may represent suitable drug targets for HF. For instance, repurposing the MIF antagonist Iguratimod may lower the risk of CAD and HF. Among the other approved drugs, FLT4 inhibitors may lower the risk of HF, but long-term therapy needed to treat HF is a limiting factor as this class of drugs is associated with a high rate of side effects [55–58]. Though further follow-up studies are needed, some of the blood candidate molecules were involved in pathways, which could be targeted in HF. For instance, glycosylation and the activin pathways may offer novel opportunity for the treatment of HF. For instance, bimagrumab, a monoclonal antibody, is an antagonist of the activin type II receptors (ActRII) [59]. In patients with type 2 diabetes and obesity, a phase 2 study has shown that bimagrumab increased the lean mass and enhanced insulin sensitivity [60]. In addition, the development of new fusion proteins between a target and a peptide, such as the Fc domain, increase the biological activity of the target-protein [61]. As a case in point, considering the protective effect of secreted BAG3 on the risk of HF, the functionalization of this protein could represent a therapeutic opportunity.

The present study has some limitations. MR is subject to horizontal pleiotropy [9]. As such, some IVs may be associated with the outcome through unknown pathways, which are not related to the exposition. However, we have implemented several measures to decrease the risk of horizontal pleiotropy. The selection of *cis*-instruments instead of *trans*-instruments is known to lower the risk of pleiotropy [9, 62]. In addition, both the Cochran’s Q test for heterogeneity and the Egger-intercept tests provide further assessment of pleiotropy [18, 19]. Finally, the implementation of the weighted median MR, which is resistant to invalid instruments (i.e. IVs related to the outcome through another process not related to the exposition) also provided robustness to the results [20].

## Conclusion

By using MR, we identified circulating proteins causally associated with HF. These proteins highlighted molecular pathways playing a key role in the development of this disorder. Integrative analysis showed that proteins were involved in several interactions where they were acting as hub molecules. Some of the molecules or pathways could be targeted for drug repurposing or the development of new small molecules or biologics in order to treat HF.

## Methods

### Genetic associations for heart failure

Summary statistics from GWAS data of a meta-analysis including 47,309 HF cases and 930,014 controls were downloaded for analyses (HERMES) [3]. The GWAS meta-analysis included participants of European ancestry from 26 cohorts. Cases included clinical cases of HF from any aetiology. GWAS meta-analysis was adjusted for age, sex and principal components and included 8,281,262 genetic variants.

### Mendelian randomization

To derive the exposures, protein expression quantitative trait loci (pQTL) were leveraged from the INTERVAL study, which included data for 2,965 different blood proteins measured in 3,301 individuals [14]. Blood proteins were measured using the SOMAscan assay. The study reported 10,572,788 genetic variants. Two-sample MR using the INTERVAL and (exposure) and HERMES (outcome) studies was carried out by using at least 3 *cis*-instrumental variables (at *P* < 1E-03 and *P* < 1E-05) selected within a window of ± 500 kb around the transcription start site of the candidate gene. Independent (*r*^2^ < 0.1) instrumental variables (SNPs) were identified with PLINK1.9 based on genotypes from European populations from the 1000 Genome project. Horizontal pleiotropy was evaluated by using the Cochran’s Q test and the Egger-intercept test. We performed inverse variance weighted MR and as sensitivity analyses we used the weighted median MR, which allows the use of up to 50% of invalid instruments. F-statistic [15–17] was calculated for each IV using the formula β^2^/SE^2^. Multivariate MR using HF as the outcome was performed by correcting the exposition for selected cardiovascular traits (blood pressure and CAD). MR analyses were performed by using the Mendelian Randomization package. Multiple test correction was performed, and significance was established at FDR < 0.05. FDR was calculated by using the R package multtest with the Benjamini and Hochberg test. The Wald ratio was calculated when only one IV with a *P* value < 1E-05 was available. The Wald ratio was calculated with the TwoSampleMR library in R.

### Reverse Mendelian randomization

Two sample reverse MR was carried out by using the HERMES study as the exposure and the blood proteins from the INTERVAL study as the outcome. IVs were selected by using a window of 500 kb at risk loci and by using the lead GWAS significant variant (*P* < 5E-08). In total, we leveraged 11 independent *cis*-instrumental variables in the HERMES study. We performed inverse variance weighted MR and the weighted median MR. Horizontal pleiotropy was evaluated using the Egger-intercept test and the Cochran’s Q test.

### Replication in deCODE

For replication we used the deCODE study [21], which included data for 4,719 different blood proteins measured in 35,559 Icelandic individuals. Blood proteins were measured by using the SOMAscan version 4 assay. The study reported 27,2 million genetic variants. Analysis was conducted for the nineteen causal candidate proteins and MR was performed as described above for the INTERVAL study.

### Enrichment analyses

To perform enrichment analyses, we downloaded data from the Comprehensive GlycoEnzyme Database (GlycoEnzDB) [63] and the ligand-receptor repository from Shao et al. [24]. Hypergeometric test was performed by using R. Pathway and gene ontology enrichments were performed by using enrichR [64, 65]. EnrichR Pathways and Gene Ontology were generated on 2021/10/20. The Comprehensive GlycoEnzyme Database was downloaded on 2021/10/22.

### Network analyses

Candidate causal blood proteins were used as seeds to generate a network based on data from InnateDB, which includes more than 19,800 curated protein interactions [25]. Edge list of association pairs was analyzed by using NetworkAnalyst [66]. We identified hub nodes (genes) as those with a degree ≥ 90^th^ percentile. Network of protein / protein interactions was generated on 2021/10/20 using Generic PPI / IMEx parameters.

### Cross-trait analysis

GWAS for HF was evaluated with the Cross-Phenotype Analysis of GWAS database (iCPAGdb) [27]. iCPAGdb uses ancestry LD-specific association data across 3,793 traits-disorders, which were selected from the NHGRI-EBI GWAS catalog to compute cross-phenotype enrichment analyses. iCPAGdb reports pairwise traits and shared signal. Output data are reported as Fisher exact test with adjustment (Benjamini–Hochberg and Bonferroni) and the Chao-Sorenson similarity index. Results of cross-trait analysis were generated on 2021/10/21.

### Multi-trait Mendelian randomization analysis

Multi-trait Inverse Variance Weighted MR were performed by using data of pQTL from the INTERVAL study (exposure) and 31 traits-disorders (outcomes). Traits were selected from 7 disease categories (atopic, autoimmune, cancer, cardiovascular, infectious, metabolic and neurologic). Data were downloaded from the NHGRI-EBI GWAS catalog and UK Biobank data previously processed by the Neale lab (see Data availability). Association from the MR analyses were deemed significant after applying the Bonferroni correction (*P* < 1.61E-03, 0.05/31). Results were illustrated as a balloon plot. Graphs were generated with ggplot2 in R.

### Drug target analysis

We assessed the druggability of each protein candidates by using the following resources: Therapeutic Target Database (TTD) [31], Drug Gene Interaction Database (DGIdb) [32] and Open Targets [33, 34]. For each repository, we reported the drug-gene pairs by using approved and non-approved drugs. In the Open Targets database, we also reported the tractability index for the development of antibodies. Drug target analysis were generated on the 29 to 30/11/2021 using DGIdb v 4.2.0; Open targets v 21.11 and TTD (update of 2021/09/29).

## Supplementary Information

**Additional file 1: Suppl. Figure 1.** Glycosylation pathway for Sialyl-lewis x. *FUT3* and *ST3GAL6* encode for fucosyl transferase 3 and ST3 beta-galactoside alpha-2,3-sialyltransferase 6, respectively. They are involved in the synthesis of sialyl Lewis x. **Suppl. Figure 2.** Gene Ontology of ligand-receptor pairs derived from the blood candidate proteins. Ligand-receptor pairs were identified through publicly available database[24]. Gene Ontology enrichment analysis performed using the 43 identified ligands/receptors pairs and the GO molecular function database.

**Additional file 2: Suppl. Table 1.** Mendelian randomization INTERVAL (*P* < 1E-03). Results of MR analysis for the nineteen causal candidate proteins in INTERVAL. Table includes: the aptamer and corresponding id code along with the protein name; the number of SNPs used as IVs in MR for each protein; the median, minimum and maximum F-statistics value for IVs; the beta, standard error and *p*-values for Inverse Variance Weighted and Weighted Median methods; heterogeneity tests (Cochrane’s Q test and intercept Egger test). **Suppl. Table 2.** Mendelian randomization INTERVAL (*P* < 1E-03) (all results). Results of MR analysis for all the proteins with enough IVs in INTERVAL. Table includes: the aptamer and corresponding id code along with the protein name; the number of SNPs used as IVs in MR for each protein; the median, minimum and maximum F-statistics value for IVs; the beta, standard error and *p*-values for Inverse Variance Weighted and Weighted Median methods; heterogeneity tests (Cochrane’s Q test and intercept Egger test). **Suppl. Table 3.** Mendelian randomization INTERVAL (*P* < 1E-05). Results of MR analysis for the nineteen causal candidate proteins in INTERVAL. Table includes: the aptamer and corresponding id code along with the protein name; the number of SNPs used as IVs in MR for each protein; the median, minimum and maximum F-statistics value for IVs; the beta, standard error and *p*-values for Inverse Variance Weighted and Weighted Median methods; heterogeneity tests (Cochrane’s Q test and intercept Egger test). **Suppl. Table 4.** Mendelian randomization INTERVAL (Wald ratio; *P* < 1E-05). Results of mendelian randomization with Wald ratio, using INTERVAL as the exposure. Wald ratio was calculated using the most significant SNP for proteins not presenting enough instruments for previously described two-sample mendelian randomization. Table includes: the aptamer and corresponding id code along with the protein name; the corresponding lead SNP identification; the beta, standard error and *p*-values for the GWAS outcome, the GWAS pQTL and for the Wald ratio. **Suppl. Table 5.** Reverse Mendelian Randomization for the nineteen causal candidates. Results of reverse MR for the nineteen causal candidate proteins identified in the study. Table includes: the aptamer and corresponding id code along with the protein name; the number of SNPs used as IVs in MR for each protein; the beta, standard error and *p*-values for Inverse Variance Weighted and Weighted Median methods; heterogeneity tests (Cochrane’s Q test and intercept Egger test). **Suppl. Table 6.** Mendelian randomization deCODE (*P* < 1E-03). Results of mendelian randomization for all nineteen causal candidate proteins, using deCODE [21] as the exposure. Table includes: the aptamer and corresponding id code along with the protein name; the number of SNPs used as IVs in MR for each protein; the median, minimum and maximum F-statistics value for IVs; the beta, standard error and *p*-values for Inverse Variance Weighted and Weighted Median methods; heterogeneity tests (Cochrane’s Q test and intercept Egger test). **Suppl. Table 7.** Mendelian randomization deCODE (*P* < 1E-05). Results of mendelian randomization for all nineteen causal candidate proteins, using deCODE [21] as the exposure. Table includes: the aptamer and corresponding id code along with the protein name; the number of SNPs used as IVs in MR for each protein; the median, minimum and maximum F-statistics value for IVs; the beta, standard error and *p*-values for Inverse Variance Weighted and Weighted Median methods; heterogeneity tests (Cochrane’s Q test and intercept Egger test). **Suppl. Table 8.** Mendelian randomization deCODE (Wald ratio; *P* < 1E-05). Results of mendelian randomization with Wald ratio, using deCODE [21] as the exposure. Wald ratio was calculated using the most significant SNP for protein not presenting enough instruments for previously described two-sample mendelian randomization. Table includes: the aptamer and corresponding id code along with the protein name; the corresponding lead SNP identification; the beta, standard error and *p*-values for the GWAS outcome, the GWAS pQTL and for the Wald ratio. **Suppl. Table 9.** Ligand-receptor pairs generated from causal blood candidate proteins. Ligand-receptor interactions for causal candidate blood proteins by using the comprehensive repository reported by Shao et al. [24]. Bold are causal candidate blood proteins. **Suppl. Table 10.** Gene Ontology (molecular function) for ligand-receptor pairs generated from the causal candidate proteins. Enrichment of all ligand-receptor pairs in Gene Ontology (GO Molecular Function). **Suppl. Table 11.** Network nodes (proteins) and degree. Degree for each protein in the network. **Suppl. Table 12.** Pathway enrichment (KEGG) for the network. Summary results of enrichment for all proteins in the network by using the Kyoto Encyclopedia of Gene and Genomes (KEGG) database. **Suppl. Table 13.** Cross-phenotype association analysis of HF by using iCPAG. Summary of iCPAG results. Trait 1 is the GWAS data from the HERMES study; Trait2 is the trait-disorder, which is compared to trait1 for sharing a similar genetic architecture. Reported in the table: *p*-value, FDR and Bonferroni adjusted *p*-value for the Fisher’s exact test; the Chao-Sorensen similarity index between trait 1 and trait 2; the list of SNPs in common between trait 1 and 2; links to corresponding experimental factor ontology (EFO) in EMBL-EBI database. **Suppl. Table 14.** Multi-trait MR. Table summarizing the 31 traits and diseases used in the multi-trait MR. **Suppl. Table 15.** Results of multivariable MR corrected for cardiovascular traits. Summary of multivariable MR and univariate MR for BAG3, MIF and APOA5. Univariate exposure is HF, whereas multivariate exposure is HF corrected for the selected trait in parenthesis. Reported are: estimate (beta), se (standard error) and *p*-value. DBP: diastolic blood pressure; SBP: systolic blood pressure; CAD: coronary artery disease. **Suppl. Table 16.** Therapeutic Target Database (TTD) for the causal candidate proteins. Summary results of druggable genome in the Therapeutic Target Database (TTD) for the causal candidate proteins. Reported are: the gene symbol and name; target type; disease for which there is an indication; drugs associated to the target. NA: not available. **Suppl. Table 17.** Drug Gene interaction Database (DGIdb) for the causal candidate proteins. Summary of druggable genome in the Drug Interaction database (DGIdb) for the causal candidate proteins. Reported are: the gene symbol; drug associated with the target; sources and pmids from the National Library of Medicine. **Suppl. Table 18.** Open Targets for the causal candidate proteins. Summary of druggable genome in the Open Targets for the causal candidate proteins. Reported are: gene symbol; drugs associated with the target; drug type (sm: small molecule, ab: antibody); category ab is the prediction confidence that the target is tractable for the development of an antibody.

## Data Availability

All data are available in the manuscript including in supplementary tables and figures. Summary statistics for GWAS HF and GWAS pQTL INTERVAL are publicly available. URLs are provided in the manuscript Data availability section.

## Abbreviations

CAD: Coronary Artery Disease
DBP: Diastolic Blood Pressure
FDR: False Discovery Rate
GO: Gene Ontology
GWAS: Genome Wide Associated Study
HF: Heart Failure
IVs: Instrumental variables
IVW: Inverse Variance Weighted
LD: Linkage Disequilibrium
LVF: Left Ventricular function
MR: Mendelian Randomization
Multi-trait MR: Multi trait Mendelian Randomization
Multivariable MR: Multivariable Mendelian Randomization
pQTL: Protein Quantitative Trait Loci
SBP: Systolic Blood Pressure
SNP: Single Nucleotide Polymorphism
