# Supplementary figures and images for: Mendelian randomization of circulating proteome identifies actionable targets in heart failure
Source: BMC Genomics. 2022 Aug 13;23:588. doi: 10.1186/s12864-022-08811-2 (PMC9375407; doi:10.1186/s12864-022-08811-2)

## **Supplementary Material**

Figure S1.

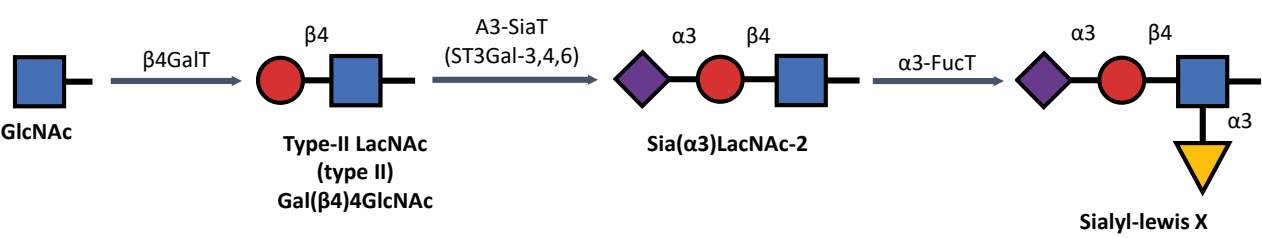

Figure S2.

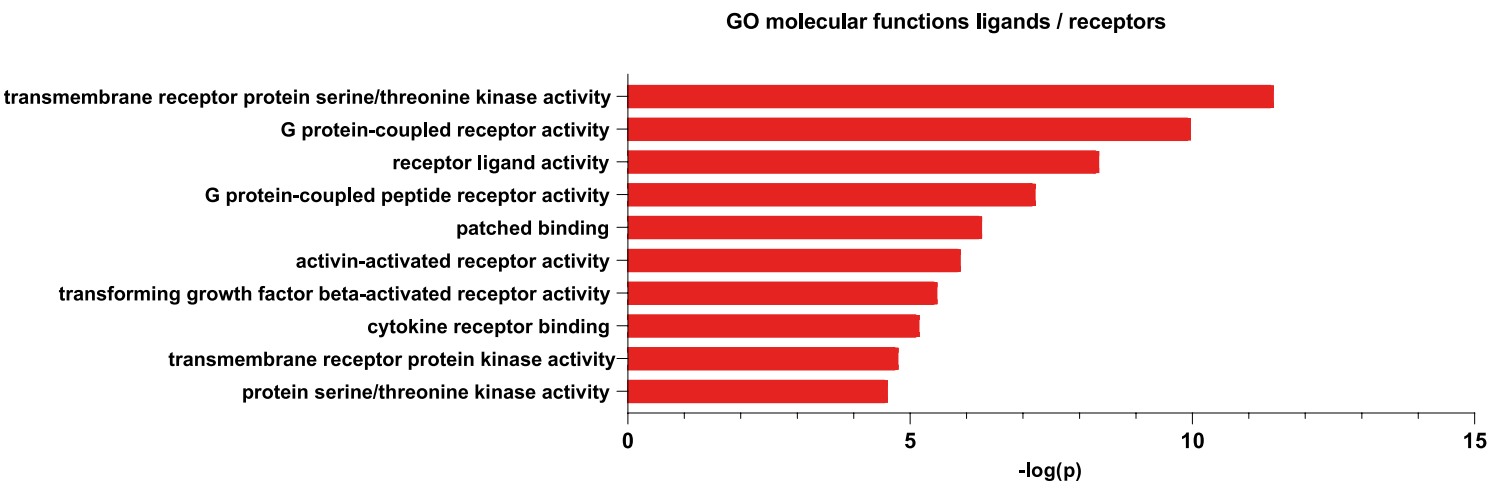

Supplement: Supplementary file 1 — Additional file 1: Suppl. Figure 1. Glycosylation pathway for Sialyl-lewis x. FUT3 and ST3GAL6 encode for fucosyl transferase 3 and ST3 beta-galactoside alpha-2,3-sialyltransferase 6, respectively. They are involved in the synthesis of sialyl Lewis x. Suppl. Figure 2. Gene Ontology of ligand-receptor pairs derived from the blood candidate proteins. Ligand-receptor pairs were identified through publicly available database[24]. Gene Ontology enrichment analysis performed using the 43 identified ligands/receptors pairs and the GO molecular function database. [file 12864_2022_8811_MOESM1_ESM.pdf]
